# Supplementary material for: The benefits of exploring a large scenario space for future energy systems
Source: Nat Commun. 2025 Dec 23;17:873. doi: 10.1038/s41467-025-67593-9 (PMC12827242; doi:10.1038/s41467-025-67593-9)
Supplement: Supplementary file 1 — Supplementary Information [file 41467_2025_67593_MOESM1_ESM.pdf]

Supplementary information

for the article “The benefits of exploring a large scenario space for future energy systems”

Ulrich Frey[1,2,*], Karl-Kiên Cao[1], Shima Sasanpour[1], Jan Buschmann[1] & Thomas Breuer[3]

[1] German Aerospace Center (DLR), Institute of Networked Energy Systems, Curiestr. 4, 70563 Stuttgart, Germany

[2] University of Graz, Department of Environmental Systems Sciences, Merangasse 18, 8010 Graz, Austria

[3] Forschungszentrum Jülich GmbH, Wilhelm-Johnen-Straße, Jülich Supercomputing Centre, 52428 Jülich, Germany

[*] Corresponding authors: karl-kien.cao@dlr.de, ulrich.frey@uni-graz.at

List of Supplementary Figures

Supplementary Figure 1. Pairplots for all indicators for scenario group E6 2

List of Supplementary Tables

Supplementary Table 1. Range of parameter uncertainties.. 2
Supplementary Table 2. Overview of the scenario groups.. 5
Supplementary Table 3. Overview of compute and data resource usage 5
Supplementary Table 4. Descriptive Statistics for the seven indicators 6
Supplementary Table 5. Differences in results and indicators in %... 6
Supplementary Table 6. Pairwise t-tests for 7 core indicators for method choice (I.) 8
Supplementary Table 7. Pairwise t-tests for 7 core indicators for method choice (II.) 8
Supplementary Table 8. Pairwise t-tests for 7 core indicators for method choice (III.) 8
Supplementary Table 9. Pairwise t-tests for 7 core indicators for method choice (IV.) 9
Supplementary Table 10. Pairwise t-tests for 7 core indicators for method choice (V.) 9
Supplementary Table 11. Mean capacity of technologies per scenario ensemble 9
Supplementary Table 12. Standard deviations of capacities per scenario ensemble 10

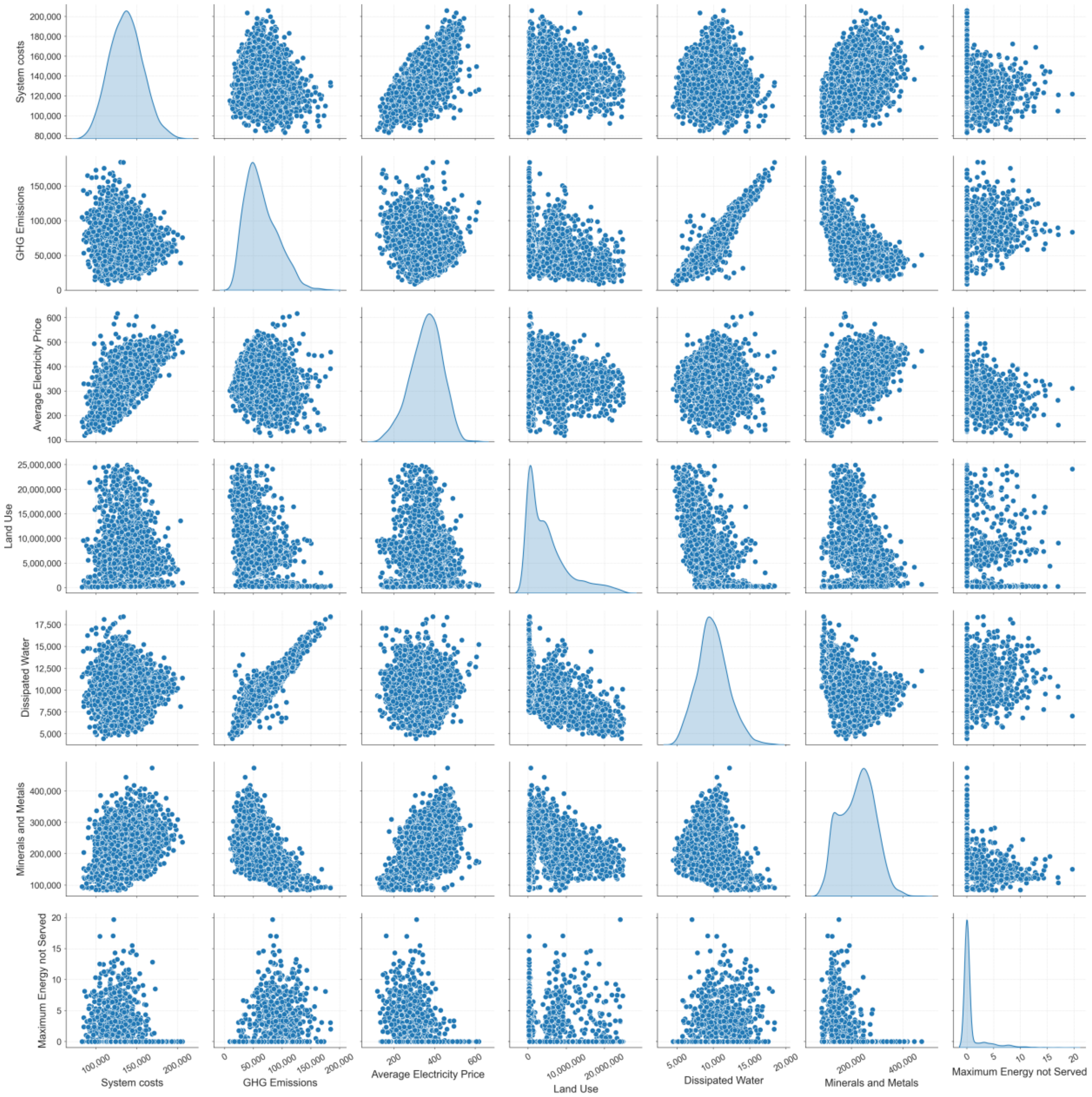

Supplementary Figure 1. Pairplots for all indicators for scenario group E6 (uniform distribution, medium-size instance, brownfield optimization, 24 weather years and no network unavailability). The indicator for the greenhouse-gas emissions and the indicator for dissipated water show a high correlation.

Supplementary Table 1. Range of parameter uncertainties used for sampling in Monte Carlo simulation.

Parameter	Technology/ commodity	Unit	Minimum	Maximum	Median
**Price**	CO_2	€/t	100	360	200
	Coal	€/kWh	0.008	0.014	0.011
	Natural gas	€/kWh	0.021	0.300	0.029
	Lignite	€/kWh	0.018	0.018	0.018
	Biomass	€/kWh	0.012	0.06	0.036
**Investment cost**	OCGT	€/kW	299.59	568.24	452.98
	CCGT	€/kW	573.95	1194.76	756.58
	Coal power plant	€/kW	1416.78	1696.00	1448.71
	Lignite power plant	€/kW	1304.26	1956.40	1625.78
	Photovoltaic	€/kW	215.88	983.87	346.94

	Wind onshore	€/kW	869.95	2399.74	1159.93
	Wind offshore	€/kW	1605.26	5202.07	2526.98
	Hydro run-of-river	€/kW	1040.37	3106.95	1664.71
	Biomass power plant	€/kW	2589.12	7411.70	4161.09
	Li-ion battery (converter)	€/kW	17.15	25.83	25.83
	Pumped hydro (converter)	€/kW	34.38	662.82	402.17
	Li-ion battery (storage)	€/kWh	75.17	556.97	213.27
	Pumped hydro (storage)	€/kWh	5.15	97.12	13.01
	HVDC (land)	€/kW	0.30	1.17	0.58
	HVDC (sea)	€/kW	1.42	6.21	2.70
	HVAC	€/kW	0.47	1.30	1.08
	HVDC converter station	€/kW	679.45	905.94	679.45
**Fixed O&M cost**	OCGT	€/kW	9.84	17.29	14.61
	CCGT	€/kW	17.22	35.84	27.76
	Coal power plant	€/kW	20.71	56.20	45.19
	Lignite power plant	€/kW	53.80	64.85	62.14
	Photovoltaic	€/kW	6.87	22.63	12.43
	Wind onshore	€/kW	20.71	55.93	32.93
	Wind offshore	€/kW	32.11	195.84	89.51
	Hydro run-of-river	€/kW	15.43	54.10	15.54
	Biomass power plant	€/kW	62.55	351.24	168.91
	Li-ion battery (converter)	€/kW	0.26	0.39	0.39
	Pumped hydro (converter)	€/kW	0.52	19.88	4.83
	Li-ion battery (storage)	€/kWh	1.13	8.36	3.20
	Pumped hydro (storage)	€/kWh	0.08	1.46	0.74
	HVDC (land)	€/kW	0.002	0.065	0.03
	HVDC (sea)	€/kW	0.006	0.006	0.006
	HVAC	€/kW	0.033	0.068	0.037
	HVDC converter station	€/kW	6.79	9.97	7.47
**Amortiza-tion time**	OCGT	years	30	35	30
	CCGT	years	30	35	30
	Coal power plant	years	40	40	40
	Lignite power plant	years	40	40	40
	Photovoltaic	years	20	35	27.5
	Wind onshore	years	18	25	22.5
	Wind offshore	years	18	30	22.5
	Hydro run-of-river	years	30	60	40
	Biomass power plant	years	20	30	25
	Li-ion battery (converter)	years	16.6	25	21.1
	Pumped hydro (converter)	years	20	80	40
	Li-ion battery (storage)	years	7.7	30.6	18.4
	Pumped hydro (storage)	years	40	84	75
	HVDC (land)	years	40	80	60
	HVDC (sea)	years	40	50	40
	HVAC	years	40	50	40
	HVDC converter station	years	20	50	30
**Variable O&M cost**	OCGT	€/kWh	0.000	0.001	0.001
	CCGT	€/kWh	0.000	0.051	0.002
	Coal power plant	€/kWh	0.000	0.038	0.002
	Lignite power plant	€/kWh	0.000	0.005	0.002

	Photovoltaic	€/kWh	0.000	0.000	0.000
	Wind onshore	€/kWh	0.000	0.021	0.016
	Wind offshore	€/kWh	0.000	0.02	0.008
	Hydro run-of-river	€/kWh	0.000	0.000	0.000
	Biomass power plant	€/kWh	0.001	0.008	0.004
	Li-ion battery	€/kWh	0.000	0.000	0.000
	Pumped hydro	€/kWh	0.001	0.001	0.001
**Efficiency**	OCGT	-	0.43	0.455	0.442
	CCGT	-	0.58	0.63	0.621
	Coal power plant	-	0.45	0.509	0.484
	Lignite power plant	-	0.47	0.491	0.491
	Biomass power plant	-	0.07	0.43	0.293
	Li-ion battery	-	0.93	0.98	0.975
	Pumped hydro	-	0.70	0.84	0.80
	HVDC	-	0.986	0.986	0.986
**Lifetime**	OCGT	years	30	35	30
	CCGT	years	30	35	30
	Coal power plant	years	40	40	40
	Lignite power plant	years	40	40	40
	Photovoltaic	years	20	35	27.5
	Wind onshore	years	18	25	22.5
	Wind offshore	years	18	30	22.5
	Hydro run-of-river	years	30	60	40
	Biomass power plant	years	20	30	25
	Li-ion battery (converter)	years	16.6	25	21.1
	Pumped hydro (converter)	years	20	80	40
	Li-ion battery (storage)	years	7.7	30.6	18.4
	Pumped hydro (storage)	years	40	84	75
	HVDC	years	40	50	40
	HVAC	years	40	80	60
**Annual potential**	Biomass, Germany	TWh	207.00	553.14	380.07
**Annual demand**	Power, Germany	TWh	459.00	841.00	535.40

The minimum, maximum and median values are obtained from a broad literature study [1]. Since the CO_2-price was rather low concerning the currently necessary goals in CO_2-mitigation, we added the price assumptions of the Kopernikus project [2]. Furthermore, we considered a higher maximum fuel price for natural gas due to the gas supply shocks in Germany in 2022.

Supplementary Table 2. Overview of the scenario groups used in the analysis of the methodological choices.

Name	Distribution	Resolution/ Size	Brown-field	Number scenarios	Number of weather years
**A 1**	Normal	Large	Yes	20	1
**A 2**	Normal	Large	Yes	80	1
**B 2**	Normal	Small	No	200	1
**C 1**	Uniform	Very large	Yes	300	24
**D 1**	Normal	Small	Yes	1300	24
**D 2**	Normal	Small	No	1300	24
**D 3**	Uniform	Small	Yes	1300	24
**D 4**	Uniform	Small	No	1300	24
**E 1**	Uniform	Medium	Yes	2000	1
**E 2**	Uniform	Medium	Yes	1600	1
**E 3**	Normal	Medium	Yes	2000	1
**E 4**	Normal	Medium	Yes	1300	24
**E 6**	Uniform	Medium	Yes	3000	24
**E 7**	Uniform	Medium	No	1300	24
**G 1**	Uniform	Large	Yes	700	24
**O 1**	Uniform	Medium	Yes	100	24
**O 2**	Uniform	Medium	Yes	100	24
**O 3**	Uniform	Medium	Yes	100	24
**O 4**	Uniform	Medium	Yes	100	24
**O 5**	Uniform	Medium	Yes	100	24

Supplementary Table 3. Overview of compute and data resource usage.

Name	Core hours	Data [TB]	Directories	Files
**A1**	330,005	0.4	442	17404
**A2**	1,078,845	0.8	992	42213
**B2**	14,851	0.3	4127	52646
**C1**	3,159,967	4.5	5464	247700
**D1**	77,289	1.8	27414	338478
**D2**	104,591	1.8	27419	338534
**D3**	83,305	1.8	27409	338461
**D4**	99,833	1.8	27404	338454
**E1**	114,402	1.2	34105	214180
**E2**	551,316	4.2	33720	416465
**E3**	109,002	1.2	34106	213791
**E4**	331,234	3.4	27410	338395
**E6**	632,148	7.9	63193	780518
**E7**	393,531	3.4	27417	338504
**G1**	1,497,357	4.4	14648	181890
**O1-5**	134,738	1.3	10495	130002
**SUM**	8,712,414	40.2	365,765	4327635

Supplementary Table 4. Descriptive Statistics for the seven indicators.

Variable name	% complete	Mean	SD
CO_2-emissions	0.98	58973.56	24431.92
System cost	0.98	126395.7	17647.24
Max. energy not served	0.98	15448.17	3199.05
Land use	0.99	4262727	4940923
Use of minerals & metals	0.99	375655.7	93151.07
Water use	0.99	11633.15	2351.91
Mean electricity price	0.98	310.33	77.68

Supplementary Table 5. Differences in results and indicators in % compared to the reference run with medium resolution, uniform distribution, 24 weather years, brownfield.

	% difference in comparison to benchmark	Normal distribution	Green-field	Very large resolution	Large resolution	Small resolution	Weather 1 year	Outage
Capacity	Installed bio power plant capacity	-12.1	-47.8	0.5	0.1	0.6	-3.4	-0.8
	Installed closed-cycle gas turbine power plant capacity	-8.9	25.1	4.6	2.1	0.7	-3.3	-4.7
	Installed natural gas turbine power plant capacity	0.0	-19.5	0	0.0	0.0	0.0	0.0
	Installed lithium-ion battery power capacity	-40.9	5.9	-3.2	0.4	-4.2	45.3	17.2
	Installed photovoltaic power plant capacity	-26.7	4.8	-5.3	0.6	-1.0	-11.4	14.3
	Installed coal power plant capacity	0.0	-12.9	0	0.0	0.0	0.0	0.0
	Installed lignite power plant capacity	0.0	-20.3	0	0.0	0.0	0.0	0.0
	Installed wind offshore power plant capacity	-3.5	-0.1	-6.2	-4.3	0.1	-5.7	-0.3
	Installed wind onshore power plant capacity	-29.3	-25.9	7.1	3.3	2.1	-19.5	5.0
Power generation	Bio power generation	-6.0	-24.1	-2.1	-3.3	1.7	-17.4	17.8
	Closed-cycle gas turbine power generation	31.6	27.3	17.7	8.4	1.7	-21.7	-21.2
	Natural gas turbine power generation	-1.0	-20.8	17.8	8.5	4.1	-31.9	-40.2
	Lithium-ion battery power output	-40.2	4.6	-8.0	-0.6	-1.3	57.2	16.6

	Photovoltaic power generation	-23.1	3.4	-3.5	0.4	0.5	40.9	9.2
	Coal power generation	-39.7	-4.5	-3.6	-0.4	-0.2	-20.0	-3.2
	Lignite power generation	-64.6	-10.1	-3.7	0.5	-3.9	-20.8	-4.2
	Wind offshore power generation	3.8	-1.0	-6.1	-5.1	-0.8	-19.7	-3.3
	Wind onshore power generation	-34.7	-19.5	17.8	9.6	-1.6	-35.5	5.8
	Curtailment of renewable energy sources	-38.5	3.6	-6.0	-0.8	-1.8	122.7	22.4
	Grid related curtailment	-37.5	7.5	76.6	26.3	-57.8	75.1	18.3
	Energy not served (REMix)	-21.8	33.8	24.7	-12.1	-2.2	-8.3	7.5
	Energy not served (AMIRIS)	-10.7	-0.7	-0.8	-2.0	-2.8	-56.7	-9.4
	Max hourly energy not served (REMix)	-2.9	23.0	11.5	-15.7	10.2	20.3	13.4
	Reserve inadequacy	-34.2	44.0	1.4	-1.4	-1.4	-38.0	2.5
	CO2 emissions from energy generation	-23.2	3.7	4.0	3.0	-0.5	-21.1	-11.6
	System costs	-24.8	-3.2	25.4	10.9	-1.9	-8.4	0.4
	Mean electricity price	-24.7	0.2	-3.2	-0.7	-1.6	-20.1	2.9
	Herfindahl-Hirschman-Index	-2.9	8.0	-2.6	-0.8	0.8	34.9	3.4
	Shannon-Wiener-Index	-1.0	-5.3	0.9	0.3	-0.4	-12.2	-1.0
	Land use for power plants	-6.6	-23.8	-2.4	-3.5	1.1	-16.7	10.2
	Minerals and metals used for power plants	-26.9	-6.8	-1.3	0.0	0.0	7.9	8.0
	Water used for power plants	-25.6	-0.4	1.6	1.8	-0.3	-10.5	-5.1

Supplementary Table 6. Pairwise t-tests for 7 core indicators for method choice (I.): different probability distribution of input parameters (for medium-sized models).

**Indicator**	***p*-value**	***t*-value**	**Degrees of Freedom**	**Normal (mean)**	**Uniform (mean)**
CO_2 emissions	< 0.001	-20.0169	3525	49149.73	63997.19
System cost	< 0.001	-71.4797	4102	103074.8	137041
Max. energy not served	0.7227	-0.3549	2682	0.86	0.88
Land use	0.0557	-1.9146	2636	4745473	5080634
Minerals & metals	< 0.001	-35.5647	3608	163484.5	223730.4
Dissipated water	< 0.001	-44.364	3415	7394.44	9940.5
Average electricity price	< 0.001	-37.1982	2747	270.04	358.48

Supplementary Table 7. Pairwise t-tests for 7 core indicators for method choice (II.): different spatial resolution.

**Indicator**	***p*-value**	***t*-value**	**Degrees of Freedom**	**Very large (mean)**	**Small (mean)**
CO_2 emissions	0.1863	-1.3247	290	63687	66545.4
System cost	< 0.001	-20.6264	267	134434.8	171846.2
Max. energy not served	0.9495	-0.0634	304	0.97	0.98
Land use	0.6613	0.4386	304	5134814	4958912
Minerals & metals	0.5512	0.5966	298	223779.1	220872.8
Dissipated water	0.2295	-1.2041	296	9908.16	10099.5
Average electricity price	0.3078	1.0217	296	352.87	346.86

Supplementary Table 8. Pairwise t-tests for 7 core indicators for method choice (III.): different capacity expansion approaches (for medium-sized models).

**Indicator**	***p*-value**	***t*-value**	**Degrees of Freedom**	**Brownfield (mean)**	**Greenfield (mean)**
CO_2 emissions	0.0175	-2.3774	2282	63,997.19	66,373.11
System cost	<0.001	6.3611	2350	137,040.95	132,619.52
Max. energy not served	0.0156	-2.4192	2224	0.88	1.08
Land use	<0.001	6.1448	2268	5,080,634.42	3,869,684.46
Minerals & metals	<0.001	6.3128	2199	223,730.44	208,609.14
Dissipated water	0.6181	0.4985	2319	9,940.5	9,903.19
Average electricity price	0.7999	-0.2535	2094	358.48	359.24

Supplementary Table 9. Pairwise t-tests for 7 core indicators for method choice (IV.): different numbers of weather years for modeling power feed-in from renewable energies (for small-sized models).

**Indicator**	***p*-value**	***t*-value**	**Degrees of Freedom**	**24 weather years (mean)**	**Single weather year (mean)**
CO_2 emissions	<0.001	14.7055	2484	49,149.73	39,589.26
System cost	<0.001	16.5338	2497	103,074.83	96,992.73
Max. energy not served	<0.001	-4.0475	1404	0.86	1.29
Land use	<0.001	7.1609	2230	4,745,472.93	3,548,811.9
Minerals & metals	<0.001	-16.0605	2859	163,484.51	188,725.27
Dissipated water	<0.001	10.3321	2361	7,394.44	6,876.35
Average electricity price	<0.001	24.2461	2289	270.04	215.31

Supplementary Table 10. Pairwise t-tests for 7 core indicators for method choice (V.): differences between full availability and unavailability of network nodes.

**Indicator**	***p*-value**	***t*-value**	**Degrees of Freedom**	**Mean no outage**	**Mean outage**
CO2-emissions	0.3704	0.8995	107	63997.19	61612
System cost	0.7354	0.3389	104	137040.95	136277.63
Max. energy not served	0.3445	-0.9497	105	0.88	1.11
Land use	0.4188	0.8117	107	5080634.42	4659641.16
Minerals & metals	0.4826	-0.7046	106	223730.44	228335.49
Dissipated water	0.456	0.7482	107	9940.5	9792.64
Average electricity price	0.6451	-0.462	104	358.48	362.52

Supplementary Table 11. Mean capacity of technologies per scenario ensemble.

**Technology**	**D3 mean**	**D4 mean**	**O1 mean**	**E4 mean**	**E1 mean**	**E6 mean**	**G1 mean**
Biomass	10.93	5.94	10.11	9.55	10.5	10.87	10.88
CCGT	31.71	39.67	31.98	28.71	30.48	31.5	32.17
Gas	12.61	10.17	12.61	12.61	12.61	12.61	12.61
Li-ion battery	46.41	49.07	50.04	28.65	70.41	48.45	48.65
Photovoltaic	431.7	451.14	448.68	319.66	386.46	436.19	438.91
Coal	8.71	7.61	8.71	8.71	8.71	8.71	8.71
Lignite	6.62	5.27	6.62	6.62	6.62	6.62	6.62
Wind offshore	43.53	43.41	43.34	42.01	41.04	43.51	41.66
Wind onshore	68.22	52.48	68.63	47.2	53.79	66.81	69.01

Supplementary Table 12. Standard deviations of capacities per scenario ensemble.

Technology	D3 SD	D4 SD	O1 SD	E4 SD	E1 SD	E6 SD	G1 SD
Biomass	5.88	9.33	5	4.31	5.8	5.75	5.85
CCGT	11.67	17.28	11.32	9.66	11.53	11.43	11.93
Gas	0	3.65	0	0	0	0	0
Li-ion battery	28.79	30.28	29.99	20.89	34.16	29.39	29.38
Photovoltaic	199.77	216.43	188.46	127.41	158.1	200.68	198.82
Coal	0	2.89	0	0	0	0	0
Lignite	0	2.65	0	0	0	0	0
Wind offshore	6.71	7.81	6.5	8.52	9.23	6.35	8.52
Wind onshore	31.87	42.69	28.5	9.47	19.08	29.32	28.44

References

1. Hermann, H. et al. Climate protection in the electricity sector 2030. Comparison of instruments to reduce emissions. Available at https://www.umweltbundesamt.de/sites/default/files/medien/1/publikationen/2017-01-11_cc_02-2017_strommarkt_endbericht.pdf (2017).
2. Cao, K.-K. & Buschmann, J. Inputs and outputs of the UNSSEN workflow including electricity price time-series, 2025. 10.23728/B2SHARE.7DFE93339C3E4E34BF4C47F880186466.
